# Acute and 28-Day Subchronic Oral Toxicity of an Ethanol Extract of *Zingiber zerumbet* (L.) Smith in Rodents

**DOI:** 10.1155/2012/608284

**Published:** 2012-03-27

**Authors:** Chia Ju Chang, Thing-Fong Tzeng, Shorong-Shii Liou, Yuan-Shiun Chang, I-Min Liu

**Affiliations:** ^1^School of Chinese Pharmaceutical Sciences and Chinese Medicine Resources, China Medical University, Taichung 40402, Taiwan; ^2^Department of Internal Medicine, Pao Chien Hospital, Pingtung County, Ping Tung City 90064, Taiwan; ^3^Department of Pharmacy and Graduate Institute of Pharmaceutical Technology, Tajen University, Yanpu Shiang, Ping Tung Shien 90701, Taiwan

## Abstract

The objective of this study was to evaluate the acute and subacute toxicity (28 days) of the ethanol extract of *Z. zerumbet *rhizomes (EEZZ) via the oral route in Wistar rats of both sexes. In the acute toxicity study, Wistar rats were administered a single dose of 15 g kg^−1^ of body weight by gavage, and were monitored for 14 days. EEZZ did not produce any toxic signs or deaths; the 50% lethal dose must be higher than 15 g kg^−1^. In the subchronic toxicity study, EEZZ was administered by gavage at doses of 1000, 2000 and 3000 mg/kg daily for 4 weeks to Wistar rats. The subacute treatment with EEZZ did not alter either the body weight gain or the food and water consumption. The hematological and biochemical analysis did not show significant differences in any of the parameters examined in female or male groups. Necropsy and histopathological examination, did not reveal any remarkable and treatment related changes. A no-observed adverse-effect level for EEZZ is 3000 mg kg^−1^ for rats under the conditions of this study. Hence, consumption of EEZZ for various medicinal purposes is safe.

## 1. Introduction

Zingiberaceae is widely distributed throughout the tropics particularly in Southeast Asia [1]. *Zingiber zerumbet* (L) Smith, commonly known as the pinecone or shampoo ginger, is a perennial, tuberous root herb plant that has gained much interest from scientists all over the world because of its high medicinal values. This herbal plant has been cultivated for thousands of years as a spice and also for medicinal purposes, that is, as a cure for headaches, swelling, colds, ulcers, sores and loss of appetite, nausea, and even menstrual discomfort and has been introduced to many parts of the world as a rich source of compounds of phytomedicinal interest [2]. The rhizome of *Z. zerumbet *has been used to treat various ailments in Asian and Arabic traditional medicine since ancient times [3, 4]. It is used in local traditional medicine as a cure for swelling, sores, and loss of appetite. The juice of boiled *Z. zerumbet *rhizomes has been used for the treatment of worm infestation in children. *Z. zerumbet* has been shown to inhibit prostaglandin-induced paw oedema, a commonly used acute inflammatory reaction and the efficacy is equivalent to the nonsteroidal anti-inflammatory drug, mefenamic acid [5]. The ethanol extracts of *Z. zerumbet* significant antipyretic activity in Brewer's yeast induced pyrexia in rats [6]. *Z. zerumbet* is also used in herbal medicinal practice for the treatment of rheumatological conditions and muscular discomfort [7]. Furthermore, the methanol extract of *Z. zerumbet* possesses inhibitory effects on platelet-activating factor and against the Den2 virus NS2B/NS3 protease activity [8, 9].

Despite knowledge of biological activities of *Z. zerumbet *rhizomes, toxicological studies are very few; it has been documented that the aqueous extract of* Z. zerumbet* at 2000 mg/kg body weight did not cause any behavioral changes to the broiler chickens immediately after its administration indicated by normal movement and drinking behavior [10]. Although herbal medicines/dietary supplements are not covered under US-FDA drug-regulatory criteria due to the consideration of their being safe, their safety profile may not have been adequately documented. Hence, preclinical acute and subchronic toxicological evaluations using Organisation for Economic Cooperation and Development (OECD) guidelines need to be undertaken to establish safety profiles of drugs of herbal origin [11]. In the previous study, we observed that the ethanol extracts from *Zingiber zerumbet* (EEZZ) with the capacity to reduce the accumulation of visceral fat and improved hyperlipidemia in high-fat-diet-induced obese rats by increasing lipid metabolism [12]. The usability of EEZZ in human cholesterol/lipid/obesity indications could be considerable, but the toxicological study on EEZZ is limitation. The present study therefore aims at investigating the acute and subchronic oral toxicity of EEZZ applying the recommended OECD guidelines for safety or dose-dependent toxicity in rats.

## 2. Materials

### 2.1. Plant Material and Extraction

*Z. zerumbet *rhizomes were purchased from a local market in Dongshan District (Tainan City, Taiwan) during October 2010. Macroscopic and microscopic examinations, as well as thin-layer chromatography and high-performance liquid chromatography, were used to confirm the authenticity of the plant material provided (this analysis was performed by Dr. Hong T. Y., Department of Biotechnology, Collage of Pharmacy and Health Care, Tajen University). Random amplified polymorphic DNA analysis of the* Z. zerumbet *rhizomes supplied was also performed to identify DNA polymorphisms. The voucher specimen (Lot No.20101018) has been deposited in our laboratory. Extraction was performed by maceration and air dried, and 5 kg of pulverized *Z. zerumbet* rhizomes was added to 10 L of 95% ethanol at room temperature for 7 days and was occasionally shaken. The ethanol extract of *Z. zerumbet *rhizomes (EEZZ) was evaporated to dryness under reduced pressure for the total elimination of alcohol, followed by lyophilization, yielding approximately 575 g of dry residue (w w^−1^ yield: 11.5%). EEZZ was kept at −20°C until use and suspended in distilled water.

### 2.2. Phytochemical Screening

 To determine the chemical constituents, qualitative phytochemical screening of EEZZ was carried out following standard procedures routinely [13] and revealed alkaloids (Meyer and Dragendoff's test), tannins (FeCl_3_ test), saponins (frothing test), lipids (Wattman paper test), flavonoids (Schinoda's test), glycosides and polyoses (NaCl, and Fehling's solutions A and B), anthraquinones (ether-chloroform and NaOH), phenols (FeCl_3_), polyphenols (K_3_Fe(CN)_6_), and terpenoids (Liberman Burchard's test).

### 2.3. Animals

 Adult female and male Wistar rats (aged 2 months, weighing 181–199 and 185–205 g, resp.) were obtained from the National Laboratory Animal Center (Taipei City, Taiwan). They were maintained in a temperature-controlled room (25 ± 1°C) on a 12 h : 12 h light-dark cycle (lights on at 06:00 h) in the animal center (Tajen University, Pingtung County, Taiwan) and had free access to standard commercial diet and tap water. All animal procedures were performed according to the Guide for the Care and Use of Laboratory Animals of the National Institutes of Health, as well as the guidelines of the Animal Welfare Act. These studies were conducted with the approval of the Institutional Animal Care and Use Committee (IACUC) at Tajen University (approval number: IACUC 99-16; approval date: September 9, 2010).

### 2.4. Acute Oral Toxicity Study

 The acute oral toxicity study was conducted using the limit test procedure according to OECD test guidelines on acute oral toxicity test 401 [14]. Twenty (10 males and 10 females) 8-week-old Wistar rats were housed individually in hygienic metabolic cages. EEZZ was administered to rats at a daily dose of 15 g kg^−1^ of body weight. EEZZ was solved in distilled water and 10 mL kg^−1^ of the sample was fed by gavage thrice daily, the interval time of the extract administration was 8 h to mimic the medication dose frequency of human. The rats were not fed for 3 h following administration. The signs of toxic effects and/or mortality were observed carefully every 0.5 to 1 h after administration on the first day, followed by daily observation, and the body weights were recorded for consecutive 14 days. LD_50_ value was calculated following the previous method [15].

### 2.5. Subchronic Oral Toxicity Study

 The method was performed according to the OECD test guidelines with slight modifications [16]. Eight-week-old Wistar rats were housed in the same conditions as described above. The eighty animals were randomly divided into four groups containing 20 rats each (10 females and 10 males). EEZZ dissolved in distilled water was administered to groups of rats at the concentrations of 1000, 2000, and 3000 mg kg^−1^ by gavage of 10 mL kg^−1^ for 4 weeks. The control group received the vehicle only. The animals were observed for signs of toxicity and mortality throughout the experimental period. The weight of each rat was recorded at weekly intervals throughout the course of the study. Food and water consumption were measured three times a week. At the end of the 4-week experiment, the animals, fasted for 12 h, were sacrificed by decapitation under anaesthesia with sodium pentobarbital (30 mg kg^−1^) administered intraperitoneally. Blood was collected into two tubes: tube 1 containing EDTA was processed immediately for haematological parameters; tube 2 without additive was centrifuged at 3000 × g at 4°C for 10 min to obtain serum (stored at −20°C until analysis). The organs (kidneys, liver, lungs, heart, testes and glands annexes, ovaries, spleen, and pancreas) were weighted. Organ samples were fixed in 10% formalin for histopathological examination.

### 2.6. Hematological and Biochemical Analysis

 Hematological analysis was performed using an automatic hematological analyzer (Coulter STKS, Beckman). Parameters included red blood cell (RBC) count, white blood cell (WBC) count, hemoglobin (Hb), hematocrit (Hct), mean corpuscular volume (MCV), mean corpuscular hemoglobin (MCH), mean corpuscular hemoglobin concentration (MCHC), red cell distribution width (RDW), platelets count, and mean platelet volume (MPV). For biochemical analysis, following parameters were determined: glucose; blood urea nitrogen (BUN); creatinine; aspartate aminotransferase (AST); alanine aminotransferase (ALT); total cholesterol; triglycerides; high-density lipoproteins (HDL); amylase; gamma-glutamyl transpeptidase (GGT); total, direct and indirect bilirubin. These levels were determined using an autoanalyser (Hitachi 7080, Japan).

### 2.7. Pathological Examination

 All animals were subjected to necropsy at the end of the toxicity studies, or earlier in case of death. Necropsy was performed to analyze the macroscopic external features of the heart, liver, spleen, lungs, kidney, adrenal gland, esophagus, stomach, small intestine (fragment of 6-7 cm), hypophysis, hypothalamus, brain, and reproductive organs (uterus and ovary or testicle, prostate, epididymis, seminal vesicle, and vas deferens). These organs were carefully removed and weighed individually. Organ weights were expressed in absolute and relative terms (g and g 100 g^−1^ of body weight, resp.). Histopathological investigation was done according to methods described in literature [17]. In addition to the organs mentioned above, the pancreas, lymph node, and bladder were also detected. Briefly, tissue samples were prepared routinely and cut into 2 *μ*m slides and stained with haematoxylin-eosin, then examined using a light microscope.

### 2.8. Statistical Analysis

 Data are expressed as the mean ± standard deviation (SD) for each group of animals at the number (*n*) indicated in tables. Statistical analysis was performed with one-way analysis of variance (ANOVA). The Dunnett range posthoc comparisons were used to determine the source of significant differences where appropriate. A *P* value <.05 was considered statistically significant.

## 3. Results

### 3.1. Phytochemical Screening of EEZZ

The phytochemical screening of EEZZ revealed the presence of the following classes of chemical compounds: alkaloids, saponins, flavonoids, tannins, terpenoids, phenols, polyphenols, and sugars.

### 3.2. Acute Toxicity

 The mean body weight of female rats increased from 190.2 ± 8.2 g to 221.1 ± 9.7 g during the 14 days, and that of males increased from 194.7 ± 8.9 g to 239.7 ± 12.8 g (data not shown). No mortalities had occurred during the study and clinical observations and measurements did not indicate evidences of substance-related toxicity. After sacrifice on the 14th day, macroscopic and gross pathology observations conducted at the necropsy examination revealed no visible lesions in any animals. Thus, no evidence of acute toxicity of EEZZ in rats was found. The oral LD_50_ values for female and male rats must be greater than 15 g kg^−1^.

### 3.3. Subacute Toxicity

No toxicity signs (such as piloerection, alteration in the locomotor activity, or diarrhea) or deaths were recorded during the 4 weeks of treatment via oral route with EEZZ at doses of 1000, 2000, or 3000 mg kg^−1^. No significant differences were found between the initial and final body weight of the rats treated with EEZZ and control rats (Table 1). A similar absence of toxic effect was observed in the case of food and water consumption (Table 1).

### 3.4. Hematological and Biochemical Parameters

The hematological profile of the treated and control groups are presented in Table 2. EEZZ did not induce any significant change in the hematological parameters such as WBC counts, total leukocyte count, hemoglobin, hematocrit, total erythrocyte count, erythrocyte indices (MCV, MCH, and MCHC), platelets count and MPV (Table 2).

The biochemical profile of the treated and control groups are presented in Table 3. The plasma levels of glucose, BUN, creatinine, AST, ALT, total cholesterol, triglyceride, and HDL of rats treated with EEZZ up to 3000 mg kg^−1^ were found to be comparable with those of the control group at end of the 28 days treatment. There was no significant difference in the plasma levels of amylase, GGT and total, direct and indirect bilirubin between vehicle-treated, and EEZZ-treated rats at any dosage (Table 3).

### 3.5. Morphological Parameters

**Figure 1:**
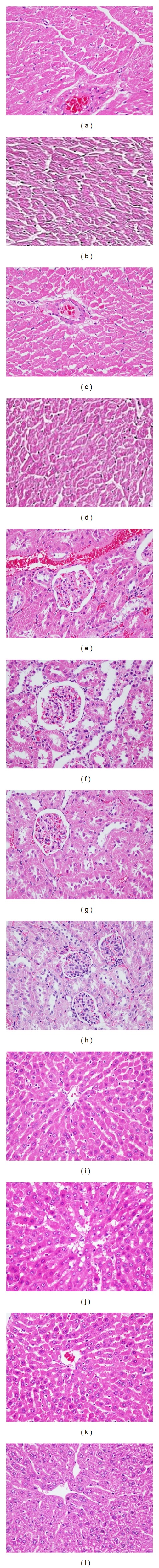
Photomicrographs of heart of control (a) and 1000, 2000, and 3000 mg kg^−1^ EEZZ-treated rat (b, c, d), kidney of control (e), and 1000, 2000, and 3000 mg/kg EEZZ-treated rat (f, g, h), and liver of control (i) and 1000, 2000, and 3000 mg/kg EEZZ-treated rat (j, k, l) stained with hematoxyline and eosin (100x).

The absolute and relative tissue weights were not altered by EEZZ treatment (Tables 4 and 5). No treatment-related macroscopic findings were observed in treated animals at necropsy. For the histological investigation, no pathological changes were observed in the hearts of animals in any group (Figure 1). Some inflammation and cell degeneration were seen in kidneys and livers of the animals treated with EEZZ; however, the changes were also detected in the control group and had no connection with the doses (Figure 1). Other organs including spleen, adrenal gland, thymus, thyroid gland, brain, uterus, testis, prostate, pancreas, absorbent gland, and bladder showed no sign of pathological changes compared with the corresponding organs of the controls.

## 4. Discussion

The results of the acute toxicity study indicated that EEZZ via oral route with the doses up to 15 g kg^−1^ did not produce any sign of toxicity or death in rats, suggesting a LD_50_ above 15 g kg^−1^ via oral route. Thus, referring to the Hodge and Stemer scale [18], the orally administered EEZZ could be considered practically nontoxic.

Repeated dose toxicity studies are conducted to evaluate the adverse effects of a test substance after prolonged use and are carried out to provide information about the possible health hazards likely to arise from repeated exposure over a relatively limited period of time including information about target organs, the possibilities of cumulative effects, and an estimate of the dose at which there is no observed adverse effect. The subacute treatment indicated that EEZZ in doses of 1000, 2000, and 3000 mg kg^−1^ per day during 28 consecutive days did not produce any deaths or clinical signs of toxicity. The doses employed in the present study represent up to 30 times more than those used as an analgesic and anti-inflammatory agent [6, 19]. A decrease in body weight would be an indicator of adverse effects [20, 21]; there were no significant changes in animal behaviour, food and water consumptions, and in body weight gain in EEZZ-treated group at any dosage. Analysis of blood parameters is relevant to risk evaluation of alterations of the haematological system in humans [22]. No significant alterations of the haematological and biochemical parameters of both male- and female-treated rats can be attributed to the plant extract.

Kidney is a sensitive organ, whose function is known to be affected by a number of factors such as drugs including phytochemicals of plant origin that ultimately lead to renal failure [23]. Assessment of possible renal damage due to EERR was made by assaying plasma urea and creatinine levels [24]. Results show no significant alteration in the plasma urea and creatinine levels due to EEZZ treatment. Moreover, there was no effect on the levels of AST and ALT, which are considered to be sensitive indicators of hepatocellular damage and within limits can provide a quantitative evaluation of the degree of damage to the liver [25]. It is reasonable to deduce, therefore, that EEZZ did not induce any damage to the liver or kidneys. This is further confirmed by the histological assessment of these organs, and the fact that plasma cholesterol levels remained unaffected, the latter being an indirect indicator of liver function [26]. No difference was observed in the weight and structure of the other organs between the control and the treated groups. Altogether, the subchronic study indicates that EEZZ ingestion did not induce detrimental changes and morphological alterations in these organs.

Since the oral dose of 3000 mg kg^−1^ per day of EEZZ administered for 28 consecutive days did not induce any biochemical, hematological, anatomical, and histopathological signs of toxicity, it can be defined as the no-observed-adverse-effect level (NOAEL) for Wistar rats of both sexes under the experimental conditions used. Information will help for future clinical studies of the medicinal safety and *in vivo* experimental studies of the pharmacological potentialities of this mode of administration of the plant medicine. However, it should be emphasized that this NOAEL was derived from a subacute study only. Since toxicity in humans cannot always be entirely extrapolated from animal studies, clinical evaluation should be performed to precisely define the safe dosage to advice in humans. For a more reliable safety evaluation performed on the basis of the acceptable daily intake concept, data on the chronic toxicity, reproductive toxicity, genotoxicity, and carcinogenicity of EEZZ would also be required.

## 5. Conclusion

In acute and subchronic toxicity studies we did not observe mortality or signs of toxicity attributable to the administration of EEZZ and no significant weight loss was registered. Therefore, the NOAEL for the acute toxicity study was 15 g kg^−1^ and for the subchronic toxicity study was 3000 mg kg^−1^. According to the dosage levels evaluated in the subchronic and acute toxicity studies, the LOAEL (Lowest Observed Adverse Effect Level) was not found for the *Z. zerumbet* rhizomes ethanol extract. Thus, the plant, at least its ethanol extract, could be considered with a wide margin of safety for oral use. Since toxicity in humans cannot always be entirely extrapolated from animal studies, clinical evaluation should be performed to precisely define the safe dosage to advice in humans.

## Figures and Tables

**Table 1:** Effects of subchronic oral administration of the ethanol extract of *Z. zerumbet *rhizomes (EEZZ) on body weight, food and water intakes in Wistar rats.

Week (W) of Treatment	Female				Male			
	Vehicle	EEZZ (mg kg^−1^ per day)			Vehicle	EEZZ (mg kg^−1^ per day)		
		1000	2000	3000		1000	2000	3000
Body weight (g)								
W1	212.97 ± 15.97	214.83 ± 13.42	215.32 ± 16.41	218.92 ± 13.56	223.61 ± 16.48	225.58 ± 14.59	229.26 ± 16.41	228.12 ± 15.92
W2	229.60 ± 14.75	224.67 ± 14.87	219.83 ± 19.26	223.97 ± 16.92	241.08 ± 15.24	235.90 ± 16.11	241.81 ± 13.88	246.37 ± 19.61
W3	245.11 ± 17.36	249.32 ± 15.96	233.51 ± 13.88	241.28 ± 15.86	257.36 ± 17.91	261.78 ± 17.26	256.68 ± 19.26	265.41 ± 20.15
W4	244.72 ± 12.63	245.21 ± 17.81	237.72 ± 14.72	243.81 ± 17.41	256.96 ± 13.08	257.47 ± 18.90	261.49 ± 14.72	268.19 ± 18.44

Food intake (g day^−1^ per rat)								
W1	22.86 ± 4.04	25.95 ± 3.01	25.29 ± 2.84	24.66 ± 3.12	24.13 ± 4.13	26.23 ± 3.47	26.36 ± 4.15	26.17 ± 4.21
W2	20.31 ± 3.69	22.25 ± 2.94	21.62 ± 3.14	20.44 ± 3.68	23.16 ± 4.45	23.32 ± 4.17	23.52 ± 3.69	23.52 ± 3.68
W3	22.40 ± 3.87	20.83 ± 3.74	21.11 ± 3.49	21.45 ± 4.95	23.21 ± 4.17	22.39 ± 3.49	22.32 ± 4.18	23.58 ± 3.95
W4	19.70 ± 3.94	20.59 ± 4.78	21.70 ± 4.32	20.97 ± 3.34	20.08 ± 3.62	21.74 ± 3.35	22.58 ± 3.93	23.14 ± 4.87

Water intake (ml day^−1^ per rat)								
W1	24.12 ± 5.62	25.63 ± 6.25	24.08 ± 6.61	24.37 ± 5.12	25.32 ± 6.18	26.38 ± 6.38	25.28 ± 7.41	25.37 ± 6.12
W2	25.09 ± 8.61	25.82 ± 5.61	24.62 ± 7.92	24.19 ± 6.83	26.34 ± 9.47	26.11 ± 6.15	25.85 ± 6.76	25.91 ± 7.38
W3	26.17 ± 5.09	25.56 ± 6.52	26.51 ± 8.12	23.82 ± 7.14	27.47 ± 5.59	26.83 ± 5.28	27.83 ± 7.31	26.27 ± 8.01
W4	24.41 ± 4.97	24.09 ± 5.18	25.41 ± 6.34	25.43 ± 7.27	25.63 ± 5.47	25.29 ± 5.18	25.41 ± 8.12	25.96 ± 6.98

Wistar rats (*n* = 10 per group) were administered ZZEE by daily gavage for 4 weeks. Data are expressed as mean ± SD.

**Table 2:** Effects of subchronic oral administration of the ethanol extract of *Z. zerumbet *rhizomes (EEZZ) on haematological parameters of Wistar rats.

Parameters	Female				Male			
	Vehicle	EEZZ (mg kg^−1^ per day)			Vehicle	EEZZ (mg kg^−1^ per day)		
		1000	2000	3000		1000	2000	3000
WBC (10^3^ *μ*L^−1^)	9.09 ± 0.59	8.69 ± 0.62	8.78 ± 0.57	8.61 ± 0.64	9.20 ± 0.68	8.90 ± 0.70	8.59 ± 0.73	8.76 ± 0.64
Lymphocytes (10^3^ *μ*L^−1^)	6.76 ± 0.40	6.66 ± 0.65	6.58 ± 0.35	7.14 ± 0.65	6.92 ± 0.75	7.36 ± 0.63	7.52 ± 0.60	7.86 ± 0.71
Erythrocytes (10^6^ *μ*L^−1^)	7.29 ± 0.07	7.69 ± 0.10	7.70 ± 0.08	7.76 ± 0.11	7.43 ± 0.11	7.34 ± 0.09	7.37 ± 0.08	7.46 ± 0.10
Hemoglobin (g dL^−1^)	16.28 ± 0.18	16.36 ± 0.17	16.29 ± 0.14	16.47 ± 0.17	17.97 ± 0.24	17.72 ± 0.18	17.48 ± 0.16	17.59 ± 0.23
Hematocrit (%)	41.17 ± 0.25	42.93 ± 0.21	43.29 ± 0.19	43.14 ± 0.21	42.53 ± 0.26	40.28 ± 0.31	40.51 ± 0.29	40.94 ± 0.23
MCV (fL)	52.14 ± 0.32	52.26 ± 0.28	50.13 ± 0.29	51.97 ± 0.32	50.35 ± 0.29	51.91 ± 0.25	52.76 ± 0.27	49.55 ± 0.23
MCH (pg)	18.86 ± 0.13	19.49 ± 0.19	20.56 ± 0.12	19.43 ± 0.12	19.85 ± 0.19	19.81 ± 0.18	19.78 ± 0.16	19.31 ± 0.17
MCHC (g dL^−1^)	35.43 ± 0.31	35.62 ± 0.42	35.57 ± 0.38	35.76 ± 0.29	35.30 ± 0.25	36.07 ± 0.33	36.04 ± 0.27	36.01 ± 0.27
RDW (%)	12.12 ± 0.15	12.15± 0.16	12.16 ± 0.14	12.15 ± 0.17	17.04 ± 0.13	17.10 ± 0.12	17.55 ± 0.13	17.57 ± 0.17
Platelets (10^3^ *μ*L^−1^)	647.54 ± 26.98	626.31 ± 25.23	665.08 ± 28.80	642.08 ± 32.44	679.54 ± 37.73	611.85 ± 39.58	619.09 ± 25.29	606.15 ± 28.31
MPV (fL)	6.08 ± 0.18	5.90 ± 0.08	5.89 ± 0.06	5.88 ± 0.08	5.48 ± 0.09	5.21 ± 0.08	5.29 ± 0.10	5.29 ± 0.07

Wistar rats (*n* = 10 per group) were administered ZZEE by daily gavage for 4 weeks. Data are expressed as mean ± SD.

**Table 3:** Effects of subchronic oral administration of the ethanol extract of *Z. zerumbet *rhizomes (EEZZ) on biochemical parameters of Wistar rats.

Parameter	Female				Male			
	Vehicle	EEZZ (mg kg^−1^ per day)			Vehicle	EEZZ (mg kg^−1^ per day)		
		1000	2000	3000		1000	2000	3000
Glucose (mg dL^−1^)	81.30 ± 7.73	84.51 ± 5.35	82.10 ± 6.19	85.88 ± 7.34	87.62 ± 5.29	89.02 ± 6.14	92.38 ± 5.21	98.62 ± 4.52
BUN (mg dL^−1^)	44.83± 2.14	45.76 ± 2.16	45.82 ± 1.98	43.51 ± 1.79	45.24 ± 1.63	46.26 ± 1.93	46.76 ± 1.72	43.52 ± 2.11
Creatinine (mg dL^−1^)	0.56 ± 0.01	0.56 ± 0.02	0.58 ± 0.01	0.55 ± 0.01	0.56 ± 0.01	0.57 ± 0.01	0.58 ± 0.01	0.56 ± 0.01
AST (U L^−1^)	149.51 ± 10.38	149.81 ± 11.51	146.24 ± 10.94	147.64 ± 12.87	143.54 ± 12.48	144.62 ± 11.29	145.83 ± 13.54	144.85 ± 10.76
ALT (U L^−1^)	71.46 ± 3.21	73.85 ± 3.19	72.25 ± 3.62	74.38 ± 2.85	70.77 ± 2.54	79.00 ± 3.47	75.58 ± 3.26	72.54 ± 2.78
Total cholesterol (mg dL^−1^)	96.50 ± 4.84	98.28 ± 5.70	100.41 ± 4.66	98.21 ± 5.50	97.77 ± 4.20	97.58 ± 5.28	93.05 ± 4.24	94.12 ± 5.15
Triglycerides (mg dL^−1^)	76.85 ± 7.65	74.23 ± 7.78	74.31 ± 8.10	73.41 ± 7.53	80.52 ± 5.67	82.45 ± 6.90	83.41 ± 8.41	82.10 ± 8.13
HDL (mg dL^−1^)	65.59 ± 3.77	61.83 ± 3.65	63.84 ± 3.24	65.72 ± 3.57	43.10 ± 2.51	40.52 ± 1.17	42.03 ± 1.89	41.30 ± 1.68
Amylase (U L^−1^)	515.26 ± 31.56	485.62 ± 37.97	482.50 ± 38.96	498.64 ± 31.67	702.64 ± 59.21	682.43 ± 29.07	714.65 ± 35.41	664.32 ± 56.11
GGT (U L^−1^)	2.47 ± 0.02	2.49 ± 0.01	2.48 ± 0.02	2.50 ± 0.01	2.53 ± 0.01	2.54 ± 0.02	2.51 ± 0.03	2.50 ± 0.02
Total bilirubin (mg dL^−1^)	0.18 ± 0.01	0.17± 0.01	0.17 ± 0.01	0.18 ± 0.01	0.16 ± 0.01	0.17 ± 0.01	0.17 ± 0.01	0.16 ± 0.01
Direct bilirubin (mg dL^−1^)	0.06 ± 0.01	0.06 ± 0.01	0.05 ± 0.01	0.05 ± 0.01	0.05 ± 0.01	0.05 ± 0.01	0.04 ± 0.01	0.05 ± 0.01
Indirect bilirubin (mg dL^−1^)	0.12 ± 0.01	0.11 ± 0.01	0.12 ± 0.01	0.13 ± 0.02	0.11 ± 0.01	0.12 ± 0.01	0.13 ± 0.01	0.11 ± 0.01

Wistar rats (*n* = 10 per group) were administered EEZZ by daily gavage for 4 weeks. Data are expressed as mean ± SD.

**Table 4:** Effects of subchronic oral administration of the ethanol extract of *Z. zerumbet *rhizomes (EEZZ) on organ weights of female Wistar rats.

Organs	Organ weight (g)				Relative organ weight (g 100 g^−1^ of body weight)			
	Vehicle	EEZZ (mg kg^−1^ per day)			Vehicle	EEZZ (mg kg^−1^ per day)		
		1000	2000	3000		1000	2000	3000
Spleen	0.445 ± 0.028	0.436 ± 0.031	0.456 ± 0.027	0.454 ± 0.027	0.182 ± 0.011	0.178 ± 0.018	0.184 ± 0.013	0.186 ± 0.012
Brain	1.111 ± 0.026	1.124 ± 0.030	1.097 ± 0.027	1.116 ± 0.031	0.454 ± 0.019	0.458 ± 0.015	0.443 ± 0.014	0.458 ± 0.016
Heart	0.853 ± 0.018	0.847 ± 0.023	0.825 ± 0.025	0.860 ± 0.019	0.349 ± 0.014	0.345 ± 0.017	0.333 ± 0.011	0.353 ± 0.009
Esophagus	0.093 ± 0.002	0.088 ± 0.003	0.093 ± 0.003	0.093 ± 0.002	0.038 ± 0.003	0.036 ± 0.002	0.038 ± 0.002	0.039 ± 0.004
Stomach	1.385 ± 0.027	1.369 ± 0.026	1.371 ± 0.031	1.379 ± 0.029	0.566 ± 0.013	0.558 ± 0.017	0.553 ± 0.015	0.566 ± 0.016
Liver	8.631 ± 0.048	8.633 ± 0.052	8.523 ± 0.046	8.651 ± 0.061	3.527 ± 0.017	3.520 ± 0.022	3.441 ± 0.019	3.548 ± 0.021
Adrenal gland	0.023 ± 0.002	0.025 ± 0.001	0.026 ± 0.002	0.029 ± 0.002	0.009 ± 0.001	0.010 ± 0.002	0.010 ± 0.003	0.011 ± 0.002
Hypophysis	0.004 ± 0.002	0.004 ± 0.001	0.004 ± 0.001	0.004 ± 0.001	0.001 ± 0.001	0.001 ± 0.001	0.001 ± 0.001	0.001 ± 0.001
Hypothalamus	0.027 ± 0.002	0.028 ± 0.001	0.029 ± 0.001	0.027 ± 0.002	0.011 ± 0.003	0.011 ± 0.002	0.011 ± 0.001	0.012 ± 0.003
Small intestine	0.516 ± 0.021	0.525 ± 0.018	0.515 ± 0.019	0.521 ± 0.022	0.211 ± 0.009	0.214 ± 0.008	0.208 ± 0.007	0.214 ± 0.009
Lungs	1.309 ± 0.053	1.278 ± 0.046	1.308 ± 0.055	1.320 ± 0.043	0.535 ± 0.017	0.521 ± 0.014	0.528 ± 0.019	0.541 ± 0.022
Kidney	0.859 ± 0.029	0.867 ± 0.034	0.874 ± 0.045	0.864 ± 0.037	0.351 ± 0.018	0.354 ± 0.019	0.353 ± 0.017	0.355 ± 0.020
Ovary	0.044 ± 0.003	0.043 ± 0.003	0.042 ± 0.002	0.042 ± 0.002	0.018 ± 0.002	0.017 ± 0.002	0.017 ± 0.003	0.017 ± 0.002
Uterus	0.546 ± 0.026	0.566 ± 0.029	0.574 ± 0.031	0.551 ± 0.039	0.223 ± 0.022	0.230 ± 0.026	0.231 ± 0.025	0.226 ± 0.025

Wistar rats (*n* = 10 per group) were administered EEZZ by daily gavage for 4 weeks. Data are expressed as mean ± SD.

**Table 5:** Effect of subchronic oral administration of the ethanol extract of *Z. zerumbet *rhizomes (EEZZ) on organ weights of male Wistar rats.

Organs	Organ weight (g)				Relative organ weight (g 100 g^−1^ of body weight)			
	Vehicle	EEZZ (mg kg^−1^ per day)			Vehicle	EEZZ (mg kg^−1^ per day)		
		1000	2000	3000		1000	2000	3000
Spleen	0.591 ± 0.038	0.571 ± 0.041	0.599 ± 0.039	0.585 ± 0.043	0.230 ± 0.012	0.222 ± 0.009	0.229 ± 0.011	0.218 ± 0.013
Brain	1.188 ± 0.015	1.197 ± 0.017	1.198 ± 0.018	1.171 ± 0.021	0.464 ± 0.022	0.466 ± 0.027	0.459 ± 0.019	0.437 ± 0.023
Heart	1.083 ± 0.048	1.072 ± 0.039	1.074 ± 0.043	1.086 ± 0.047	0.427 ± 0.009	0.330 ± 0.016	0.319 ± 0.013	0.282 ± 0.018
Esophagus	0.133 ± 0.010	0.127 ± 0.009	0.122 ± 0.011	0.130 ± 0.008	0.423 ± 0.008	0.049 ± 0.011	0.046 ± 0.009	0.049 ± 0.013
Stomach	1.438 ± 0.038	1.482 ± 0.045	1.453± 0.047	1.461 ± 0.041	0.561 ± 0.021	0.577 ± 0.019	0.556 ± 0.023	0.546 ± 0.021
Liver	9.971 ± 0.035	9.952 ± 0.043	9.986 ± 0.048	9.994 ± 0.052	3.894 ± 0.118	3.872 ± 0.153	3.826 ± 0.175	3.729 ± 0.143
Adrenal gland	0.024 ± 0.002	0.025 ± 0.001	0.021 ± 0.003	0.023 ± 0.003	0.009 ± 0.003	0.009 ± 0.002	0.008 ± 0.002	0.009 ± 0.003
Hypophysis	0.004 ± 0.001	0.004 ± 0.001	0.004 ± 0.001	0.004 ± 0.001	0.001 ± 0.001	0.001 ± 0.001	0.001 ± 0.001	0.001 ± 0.001
Hypothalamus	0.036 ± 0.001	0.035 ± 0.001	0.035 ± 0.001	0.034 ± 0.001	0.014 ± 0.002	0.014 ± 0.001	0.013 ± 0.001	0.013 ± 0.002
Small intestine	0.572 ± 0.045	0.586 ± 0.057	0.582 ± 0.063	0.597 ± 0.068	0.223 ± 0.021	0.228 ± 0.019	0.223 ± 0.017	0.223 ± 0.025
Lungs	1.435 ± 0.066	1.441 ± 0.059	1.463 ± 0.065	1.470 ± 0.078	0.381 ± 0.025	0.561 ± 0.028	0.559 ± 0.031	0.549 ± 0.032
Kidney	0.975 ± 0.053	0.967 ± 0.059	0.968 ± 0.062	0.948 ± 0.058	0.336 ± 0.015	0.346 ± 0.017	0.351 ± 0.016	0.313 ± 0.015
Epididymis	0.615 ± 0.056	0.603 ± 0.045	0.625 ± 0.038	0.620 ± 0.047	0.239 ± 0.013	0.235 ± 0.009	0.239 ± 0.010	0.232 ± 0.012
Vas deferens	0.125 ± 0.011	0.129 ± 0.009	0.131 ± 0.008	0.132 ± 0.009	0.049 ± 0.003	0.050 ± 0.005	0.050 ± 0.004	0.049 ± 0.006
Prostate	0.553 ± 0.071	0.547 ± 0.053	0.569 ± 0.064	0.565 ± 0.056	0.215 ± 0.011	0.213 ± 0.009	0.218 ± 0.013	0.211 ± 0.012
Testicle	1.618 ± 0.072	1.627 ± 0.068	1.614 ± 0.078	1.637 ± 0.059	0.629 ± 0.032	0.632 ± 0.027	0.617 ± 0.031	0.610 ± 0.025
Seminal vesicle	0.467 ± 0.022	0.471 ± 0.017	0.478 ± 0.021	0.469 ± 0.019	0.182 ± 0.007	0.183 ± 0.010	0.183 ± 0.009	0.175 ± 0.013

Wistar rats (*n* = 10 per group) were administered EEZZ by daily gavage for 4 weeks. Data are expressed as mean ± SD.
